# Preparing for partnerships in cancer care: an explorative analysis of the role of family-based caregivers

**DOI:** 10.1186/s12913-021-06611-0

**Published:** 2021-06-29

**Authors:** Reema Harrison, Madhav Raman, Ramesh Lahiru Walpola, Ashfaq Chauhan, Ursula M. Sansom-Daly

**Affiliations:** 1 Australian Institute of Health Innovation, Macquarie University, Sydney, NSW Australia; 2 School of Population Health, UNSW Sydney, Sydney, Australia; 3 School of Women’s and Children’s Health, UNSW Sydney, Sydney, Australia; 4 Behavioural Sciences Unit, Kids Cancer Centre, Sydney Children’s Hospital, Sydney, Australia; 5 Sydney Youth Cancer Service, Nelune Comprehensive Cancer Centre, Prince of Wales Hospital, Randwick, Australia

**Keywords:** Delivery of healthcare, Informal caregivers, family-based caregivers, Psychosocial support systems, Cancer care

## Abstract

**Background:**

Family-based ‘informal’ caregivers are critical to enable sustainable cancer care that produces optimal health outcomes but also gives rise to psychological burdens on caregivers. Evidence of psychosocial support for caregivers does not currently address the impacts of their role in providing clinical and health-related care for their loved ones. The present study sought to address this gap including with those from priority populations.

**Methods:**

Qualitative data was collected using focus group and interview methods. We purposively sampled caregivers identified as having a high burden of responsibility for providing clinical care including those from ethnic minority backgrounds, parental caregivers and those living rurally. Transcripts were subject to thematic analysis utilising a team-based approach.

**Results:**

Family-based caregivers included spouses (11), parents (7), children (1), siblings (1). Ten participants were from ethnic minority backgrounds and five participants were from regional or rural locations. Four resulting inter-related themes were; 1) Dual burden of providing clinical care and managing personal emotional distress; 2) Navigating healthcare partnership dynamics; 3) Developing a caregiving skillset, and 4) Unique supportive needs and barriers to access. These data provide evidence of the unique challenge of providing clinical care as part of family-based caregiving for a loved one with cancer, and the absence of support for caregivers to take up this role.

**Conclusion:**

Our findings highlight the substantial contribution of family-based caregivers to the provision of cancer care in contemporary health systems. Inadequate support for caregivers is apparent with regard to their role in providing clinical aspects of care such as medication administration and management. Support programs to prepare caregivers to provide clinical care while building capacity to manage their stressors and emotions through this challenging period may be valuable towards sustainable, person-centred care.

## Background

Family-based ‘informal’ caregivers are critical to enable sustainable cancer care that produces optimal health outcomes people who are living with or have lived with cancer worldwide. Family-based caregivers are patients’ spouses, siblings, children, parents and friends. They experience substantial emotional distress due to their loved one experiencing cancer; this experience is compounded by the responsibility of providing clinical and health-related care, which impacts caregivers’ well-being, the safety of care and care outcomes [1]. Advancements in cancer treatment and home-based supportive care means that increasingly, treatment and care is supported by families at home rather than by health professionals [2, 3]. Family-based caregivers manage and administer medications, coordinate treatment and appointments with a range of healthcare professionals and help manage diet and lifestyle supportive programs for their loved ones [4]. Providing this clinical care offers advantages for patient-centred care and sustainable delivery of care but may expose caregivers to challenges detrimental to their mental health and require psychosocial support. The psychological impacts on caregivers of providing clinical and health-related care, and the supports required for this to enable healthcare partnerships in cancer care, have not been investigated or addressed through supportive interventions [5–7].

When caregivers experience poor psychological health, people with cancer experience poorer care outcomes and psychological distress [8]. Unique features exist that distinguish the cancer caregiving experience from caregiving for other chronic health conditions. These include the potential for rapid health deterioration over a short period of time, variability in symptoms and toxicities from different multimodal therapies and the need for caregivers to monitor patients’ health status frequently to promote patient health [9]. Caregivers navigate these stressors whilst also managing their distress and concern about their loved one who has cancer [8, 10]. They can therefore carry a heavy sense of responsibility and face significant distress, particularly when their loved one’s cancer treatment trajectory is not progressing optimally or when unexpected complications in care occur [11, 12].

Within the population of informal caregivers there are some sub-groups of caregivers, who are more exposed to distress and negative health outcomes due to the cumulative impact of several sources of burden. These groups can be described as priority populations for intervention. They include parents, who are most commonly the informal caregivers for children and adolescents with cancer, are more heavily relied upon for decision-making, symptom monitoring, care coordination, and supporting their child to engage with, and adhere to, both cancer-related and supportive-care treatments than caregivers of adult patients [13]. Secondly, people with cancer who are from ethnic minority backgrounds who rely more heavily on their caregivers for advocacy and health system navigation [14, 15]. Finally, people with cancer living in regional or rural areas in which they are separated from specialist centres or services by geographical distance and therefore who rely more heavily on their informal caregivers to monitor symptoms, provide supportive and symptom-related care, and assist in travel to metropolitan treatment centres than people with cancer who reside in cities [16, 17].

Research regarding the role of caregivers focuses predominantly on their distress and worry about their loved one’s cancer diagnosis – rather than acknowledging distress that they may experience around their involvement in their loved one’s cancer clinical and health-related care and the implications of this distress for care delivery and outcomes [5]. Yet recent findings highlight that caregiver for those with cancer show significantly higher anxiety compared to the general population which is specifically correlated with coordinating clinical care and attending appointments [18]. Providing appropriate supportive programs to address this anxiety from caregiver involvement in their loved one’s cancer care requires data regarding their needs. Knowledge of the implementation factors that must be considered for supportive programs to be accessed is also critical to ensure that future supportive programs are feasible for application in a community setting [5]. The present study aimed to address this gap by exploring the role that caregivers play in providing clinical care for loved ones with cancer and their views regarding the supportive that they need to contribute to healthcare partnerships in this way, particularly for those in identified priority groups for exposure to distress and negative health outcomes.

## Methods

### Ethics approval

The study received ethics approval from the University of New South Wales Human Research Ethics Committee (HC200177).

### Design

A cross-sectional qualitative descriptive study was undertaken and reported in accordance with the Consolidated Criteria for Reporting Qualitative Studies (COREQ) guidelines to promote complete and transparent reporting of this focus group and individual interview research [19].

### Setting

Caregiver experiences across Australia were captured for those supporting loved ones through a range of cancers. The project was conducted in partnership with the following organisations: Cancer Voices, Sisters’ Cancer Support Group, Caregivers NSW, and Neuroblastoma Australia who actively support caregivers by providing information, education, raising awareness, providing supportive programs, fundraising, and research. These organisations publicised the study and there were no pre-existing relationships with participants.

### Recruitment

Recruitment was conducted via the partner organisations. Study advertisements and invitations were sent to potential participants via email distribution lists and included in social media posts. Caregivers were eligible to participate if they had experience as a past and current caregiver for one or more family members who had experienced cancer. Participants who did not have high levels of English proficiency were eligible to participate with the option to use interpreters made available where required. The project officer (MR) followed up potential participants who expressed interest by telephone to answer queries or provide additional information requested. Potential participants were then asked to read the Participant Information and Consent Form and provide their written informed consent by signing this document ahead of taking part and a copy provided to each participant. Participants had the option of attending a focus group or to select an individual interview to enable people to discuss their experiences in a context that was the most comfortable for them given the highly sensitive subject matter and diverse participant needs. As the recruitment process developed, we decided to form a focus group dedicated to culturally and linguistically diverse (CALD) populations to facilitate a synergy of ideas regarding experiences of CALD caregivers who responded to the study invitation [20]. Considering the diversity of experiences of cancer type, stage, services and geographic location amongst the population of caregivers, we did not seek to achieve data saturation but, rather to gather sufficient diversity of experience to address the research aim of capturing common experiences of caregivers in providing clinical and health-related care for loved ones with cancer and their views regarding the supportive that they need to contribute to healthcare partnerships in this way [21, 22].

### Interview schedule and focus group topic guide

The interview schedule and topic guide were developed by three researchers (RH, USD, RW) based on their clinical and research experience working with informal caregivers in cancer services [20, 23, 24]. Both documents guided the discussion through four avenues of questioning or topic areas that explored experiences of being a caregiver, experiences of psychological support, psychological support needs, and perceptions of interventional approaches that may support caregivers.

### Data collection

Three research team members with experience in qualitative research conducted the interviews and focus groups using video-conferencing software (RH, RW, MR). The research team conducted 45–60-min interviews and 60–90-min focus groups at convenient time agreed with each participant. Participants were welcomed by the facilitator and introduced to members of the group. Participants were then briefed about the study process. Participants were informed they could withdraw at any time and were informed that they had access to a clinical psychologist if they required additional support. The researchers utilised the interview or topic guide while also following up on relevant novel lines of inquiry, in addition to taking field notes. Audio recordings were transcribed verbatim in English by an accredited transcriber [19]. None of the participants required language translation.

### Analytic strategy

Thematic analysis was conducted to identify, analyse, and report patterns in the qualitative data collected across the focus group and interview transcripts collectively [25]. Transcripts were independently read by three researchers (RW, AC and MR). Once familiarised with the content of the transcript, each researcher undertook line-by-line analysis to label the data independently. Themes were generated from the initial labels and then grouped under broader categories through discussion with a fourth researcher (RH). The categories were then labelled in reference to the raw data. Interpretations of the data were resolved through multiple discussions at each stage in the analysis process.

## Results

A total of 20 caregivers participated: 10 participants across three focus groups and 10 participants in individual semi-structured interviews. Out of the ten interviewees, five were parents whose children were diagnosed with Neuroblastoma. Of the 20 participants, six were male and 14 were female. Participants in the focus groups and interviews identified being caregiver for their spouse (11), parents (1), children (7), or sibling (1). A total of 10 participants were identified as from CALD background. Of the 20, five participants were from regional or remote areas. Six participants still had care duties for cancer related treatment and management.

Four interrelated categories were developed; 1) Dual burden of providing clinical care and managing personal emotional distress; 2) Navigating healthcare partnership dynamics; 3) Developing a caregiving skillset, and 4) Unique supportive needs and barriers to access. The continuous need to adapt to meet the needs of the caregiving role was pervasive throughout the data, with relevance to all identified themes. Caregiving is inherently a diverse role which unfolds in a different way for each individual. Supportive needs are therefore dynamic and subject to change.

### Dual burden of providing clinical care and managing personal emotional distress

Providing clinical care encompassed a variety of activities from booking and driving to appointments, cooking meals, and attending personal care and hygiene care needs, to co-ordinating care, giving medications, speaking up on behalf of the patients in appointments/healthcare settings and being an interpreter between the medical team and family. These activities were completed in the context of overwhelming emotional pressure in response to a loved one’s cancer diagnosis and prognosis, creating a dual burden of managing clinical responsibility and personal emotional distress.

“I gave her medication, looking after her, taking her to the doctors, making appointments and everything” – Focus group (FG) 3 – Male, Spouse, Ethnic minority background

“I got myself involved and started talking with her oncology specialist, members of the cancer treatment team in the hospital, and just acted like a mediator between the two so that we could understand her treatment, what treatments were available, what her prognosis was.”- FG 2 - Female, Daughter, Ethnic minority background

“I had my own family to look after, and on top of that I - not that I had to, but I wanted to be there for her and her family. So they were the biggest challenges for me...” – FG3 – Female, Sibling, Ethnic minority background

Across the sample, participants identified challenges in liaising with the medical teams and being an advocate for their loved ones. Parents in particular described the additional requirement to be present for treatment and to take care of their child while in hospital, which could be in a city other than their home city. Parents described adapting to meet the needs of this broad and diverse caregiving role as needed and sometimes expressed feeling unprepared for new and additional responsibilities.

“They really rely on parents or whoever’s in the room to be able to tell them, how do you think her pain levels are, where do you think she’s at? That is something that comes from probably, as a parent, as a primary caregiver, knowing, okay, is that little cry she’s got, is that just like – she’s just annoyed but okay or is that a, ‘no, I’m really uncomfortable’. That’s hard.”- Interview, Female, Parent

As a loved one’s condition progressed, so too did the degree of clinical responsibilities that caregivers reported. Such responsibilities were often described as an additional concern, with fears of making mistakes with medication dosage and administration commonplace. Participants perceived that they faced increasing responsibility to make decisions regarding their loved one’s care.

“My main role was to - …. make sure he was eating well and resting, but also doing what he wanted to do when he wanted to do it… I was very mindful that XXX was the one who had cancer, so I was his partner and advocate to an extent, but it was his journey, and I needed to be respectful of that.” – FG 3 – Female, Spouse, Regional

“Because as the disease progressed, the caregiver’s intervention progressed and became more challenging, and was always a negotiation as to when was the next line going to be crossed? When was the next boundary extended?” – FG 1 – Male, Spouse

“You’ve got to keep a monitor on the observations…we had a drug to give every hour. I mean, it’s very, very stressful.” – Interview, Female, Parent

Absence of self-care amongst caregivers was notable in the context of an ever-present and enduring caring relationship. Participants frequently described that they had neglected their own needs as they focused on caring for their loved ones. Several participants expressed that they did not know where and how to seek help to assist them with the emotional aspect of taking care of their loved ones with cancer. Support services were often impracticable to access.

“I think, when you’ve got someone going through cancer, rightly so, there’s so much focus on the patient, - but I think the caregiver also almost needs to be treated as a separate - as a second patient” – FG 1 – Female, Daughter, Regional

“I found it difficult with my husband’s emotional state transforming to being… although strong-minded but very gentle, he became very aggressive. I was looking for help on how to deal with that transference of emotions on his part” – FG 2 – Female, Spouse, Ethnic minority background

“I was a back-up nurse…back-up for children's entertainment… it was harder coming home than being in the hospital... my husband and I fight a lot more…juggling all of that was really tiring and really really hard.” - Interview, Female, Parent

### Navigating healthcare partnership dynamics

A central role for family-based caregivers is that of partnership with health system, services, and professionals to provide care. As such, partnership dynamics between caregivers and health professionals were identified as impacting the caregiving experience. A strong partnership was characterised by participants by opportunities to ask questions at ease, feeling respected and included as part of the care team. Caregivers perceived that these partnerships were strengthened by health professionals who recognised the emotional burden of being a caregiver and provided a wider sense of support to caregivers as well as focusing on patient care needs.

“Well, we are very lucky that we had a great support, especially from the hospital, from the doctors and from friends and neighbours, very excellent neighbours…. We had a lot of moral support from everyone, and that counts.” – FG 3 – Male, Spouse, Ethnic minority background

“I think that was an excellent partnership, I really do. His doctor was amazing and they also had a nurse...like she was the interpreter between the doctor and us. I did feel like part of that partnership, they did respect that I was the caregiver.” – FG 1 – Female, Spouse

Conversely, fragmented teams, care processes and guidance contributed to pressure on caregivers to take a substantial care coordination, advocacy and a vigilant role in ensuring the correct information was transferred between healthcare providers, teams and services. It was notable that such experiences were often when entering the health care system via the emergency department, and positive experiences of multidisciplinary care teams in cancer services were commonplace.

“XX would frequently be admitted to the cancer ward at XX Hospital. The staff there knew her, knew me and there was a partnership. Frequent visits to Emergency was always a drama because there was always someone different there…… but they had an inability to listen. So you felt like you had to argue and push your way in” – FG 1- Male, Spouse

‘the communication to us of what the schedule of drugs was, was very poor and so I developed my own system at home, my own spreadsheet. Then I used my spreadsheet to correct the medical team because she'd actually been dispensed with the wrong drugs’ – Interview, Female, Parent

“really we’ve had to advocate. So we’re the ones saying we need to see the doctors. We need to refresh with this. What are we up to with treatment? It feels like we’re constantly having to keep in their faces about when are we starting the next cycle? When are we doing this? We’re talking directly to the doctors and there’s a whole crew that change all the time.” – Interview, Female, Parent, Regional/Rural

Some caregivers from ethnic minority backgrounds specifically commented on their additional role in ensuring religious and cultural requirements were known and adhered to.

‘It was more just making sure that our religious requirements were met. It was more asking for a female nurse rather than a male nurse to tend to her and little things like that.’ - FG 2 Female, Daughter, Ethnic minority background.

The partnership dynamic was placed under most pressure from interpersonal distress between caregivers and health professionals. Frustration and distress were commonly discussed by caregivers who felt that their voice was not heard, that health professionals lacked empathy, or that they were not considered as part of the care team; for example when hearing staff talking about them rather than with them. In such situations, caregivers felt reluctant to ask questions and found it therefore difficult to build a healthy partnership. Challenges in developing strong partnerships were also identified in the context of constantly changing staff or teams.

“At times, you do feel like you are the advocate. You walk a line between being the partner but also the advocate, and that's not always easy. I heard myself on more than one occasion being described by nurses as being just a little bit anxious” – FG 3 – Female, Spouse

‘The doctor in front of everybody that was there in the room having chemo on that day and there were about 15 people, he just yelled from the desk - he didn’t even come to us and chat - he just yelled over the desk that if you don’t want it go home. We went home.’ – FG 2, Female, Spouse, Regional/Rural

“They need to do a module only on how to speak to patients and how to be empathetic…. as a caregiver, as a family member, you're really concerned, and you want to know things but if you ask the questions, then you're being a burden….” – FG 3 – Female, Sibling, Ethnic minority background

### Developing a caregiving skillset

Rapid upskilling was required at the outset and throughout the course of caregiving to meet task requirements and responsibilities. Participants reported their need for training for the clinical roles required during at-home care, particularly for performing specialised tasks such as administering injections, medication via nasal pumps, or utilising feeding tubes. Support available for such skill development was limited with education generally provided initially without follow-up which was inadequate for multiple, complex or specialised tasks. Participants often discussed identifying additional organisations to access the necessary education and training.

“When your child has been so sick, you have to learn to give the medications, and you need to get that training as well, you need to know how to use their nasogastric pumps as well so the team need to teach you how to feed them give them the right rates and need to get all the gear from the dietician and making sure they are meeting their developmental milestones.” – Interview, Female, Parent, Regional/Rural

“The PEG tube and how to feed and when to do it, how to make it sterile, etcetera I didn’t know…I had to learn through other sources or go to my own doctor and have a lesson through him because I certainly wasn’t getting it from the medical professionals at the hospital” – FG 2

Participants described developing their own techniques to keep record of the medication and treatment regime and any changes to this, but also to liaise with other caregivers in the home environment. Using a notebook or a whiteboard was common and often also used to note any concerns to speak with the care providers at the next visit.

“So very quickly, we learnt skills in notetaking on a whiteboard. Whoever’s in the room writes down the stuff that’s happening...It’s crazy how many times the nurses and doctors will just come in and look at our board” –Interview, Female, Parent

“We had a notebook where we documented [medications] - as well as the chemo... to make sure that all the medications were taken at the right time, and that notebook also helped us to take notes about how he was feeling each day in response to the chemo or other things, so that we could talk about that with his oncologist.” – FG 3, Female, Spouse, Regional/Rural

### Unique supportive needs and barriers to access

Participants described a range of stressors to manage with little or no psychosocial support readily accessible. Barriers to the use of in-person psychologists or counsellors due to caring responsibilities and the desire not to discuss caregiver distress in front of their loved ones were commonly raised. Similarly, of the limited support available, requirements to attend specific sessions at specific time points were also challenging and described as an unwelcome additional pressure by several participants.

“In the early days - and also the longer you keep going through this journey – [we] could have done with someone just coming in for us, just to ask us, are you okay? You've had a hard day today; can we talk through that? To have those emotional support services for us too.” – Interview, Female, Parent, Regional/Rural

I wish someone had sat me down and said, this is what you - this is what the role is. I just had to kind of work it out for myself, but I wish someone had actually sat me down and said, this is what's involved, this is what you can expect, think about self-care of yourself - Interview, Female, Parent

“There really wasn’t [any psychological supportive available] - there are face-to-face groups but we were new to Sydney and it was just daunting enough being here, let alone having to travel out to find a group of people. I wasn’t up for that. Then with the other one in hospital, [I] just couldn’t get out [from the bedside to attend it]” – Interview, Female, Parent, Regional/Rural

Equally important to addressing the unmet need in providing psychosocial support was the need for such programs to consider the caregivers’ circumstances. The need for flexibility was a key requirement. Participants suggested content that they can listen to in their own time was preferred over written materials along with highly personalised support to address their diverse and changing support needs across the spectrum of caregiving.

“Time's an issue but you spend an enormous amount of time sitting around in hospitals and waiting, waiting…podcasts and online stuff is…probably a pretty good way of doing it.” – Interview, Female, Parent

“Something online would be great, that is why I have linked in with the Facebook online groups and it’s something about online you can pick it up when you want to access that content.” – Interview, Female, Parent

The point at which to offer or provide support was identified as a challenge. Many participants discussed not being ready to or aware of their need for support with caregiving at the outset of their journey. They reflected that as people require support at different times, supportive programs must be accessible when needed rather than at one time point across the caregiving trajectory. Consistency and continuity were further intervention attributes considered valuable by caregivers, such as being able to reach out to someone via phone call to discuss an issue and seek advice and being able to speak with that same person or group regularly when needed.

“I think the flexibility part… is the key. Because you never know what’s happening around the corner with treatment and you never know when you’re going to need that support. So to have it available when it’s needed rather than say, well you have to wait, we don’t start the next course till three weeks’ time” - FG 3 – Female, Daughter

A notable aspect of the caregiver experience for regional/rural caregivers and those located at distance from a specialist centre was the substantial lifestyle impact of caregiving, which included relocating full-or part-time from their home town and often leaving their current employment to live in a healthcare environment and/or supportive accommodation. Their home context was often referred to as influential in the way in which they may be able to access support. For example, fewer opportunities to leave the loved one’s side when leaving away from their existing support network or because of the need for parents to live apart.

“We were quickly sent from our normal life here in XXX, where we both worked bringing up a child, thrown into a cancer life…we moved into an apartment in XXX. One of us would sleep there each -only one parent could sleep in hospital, while XXX was an inpatient.” – Interview, Female, Parent, Regional/Rural

## Discussion

Informal caregivers have become an integral part of contemporary gold-standard cancer care delivery. Through a series of three focus groups and 10 interviews with 20 caregivers from diverse backgrounds, we qualitatively explored the role that informal, family-based caregivers as healthcare partners. Our data highlighted that caregivers perceived their role as complex and in a state of almost continual transition. Four key categories emerged with relevance to the clinical- and health-care related elements of their caregiving roles: 1) Dual burden of providing clinical care and managing personal emotional distress; 2) Navigating healthcare partnership dynamics; 3) Developing a caregiving skillset, and 4) Unique supportive needs and barriers to access.

While literature and supportive interventions have acknowledged the distress family members may feel in response to a loved one’s experience of a life-threatening illness such as cancer, few studies or interventions have addressed the psychological impact of the caregiving role itself in terms of the clinical, healthcare related responsibilities it can involve [5–7]. The extent to which caregivers are involved in clinical components of caregiving can evolve over time [26], just as patients’ level of dependence can also fluctuate in parallel with their changing desires and physical/psychological capacity to actively engage in their healthcare. This can be particularly the case for children, adolescent and young adult cancer patients, who face several dynamic transitions concurrently, both as cancer patients experiencing new and evolving treatment-related challenges, as well as young people developing emerging adult cognitive, socio-emotional and behavioural coping skills [27–29].Our data underscored the double distress associated with being a family member and caregiver simultaneously. It has shed light on the impact of navigating these relational challenges and boundary-crossings but also the unique challenges faced by some populations of family-based caregivers to manage this distress at extensive physical distance from their support networks, to leave their employment to be within service reach or to strive to upload religious and cultural requirements in the context of clinical care provision.

Wider evidence highlights that parents of young adults also carry substantial parental caregiving responsibility yet are isolated when entering the adult system that does not have the same family-based approach as paediatric care. These caregivers report that medical information needs constitute their highest unmet need contributing to psychological distress [30]. Given evidence of the poorer cancer care outcomes that exist for some of these sub-groups, including rural/remote and CALD communities [31, 32] it is possible that strengthening the caregiver-healthcare partnership may be key to optimising clinical outcomes.

### Clinical implications

Our data point to several clinical implications that warrant further study. While interventions exist to address caregivers’ distress and coping related to their loved one’s cancer [33–36], few interventions directly address the psychological impact of the healthcare-related aspects of cancer caregiving. Supportive requirements to attend to the practicalities of caregiving, such as medication administration may be addressed through training for caregivers in hospital and/or community settings, but psychological support is required to attend to the associated experiences of anxiety and stress. An overarching notion of the requirement for ‘meta-skills’ to enable caregiving. These meta-skills describe the psychological and communication skills needed to feel prepared for, and to successfully navigate, the spectrum of caregiving across the cancer trajectory.

Caregivers in the study sample expressed the need to negotiate with healthcare teams regarding expectations of their role and identified challenges in communicating and advocating for their loved one, in being ‘heard’ without being mistaken or perceived as overly anxious, and identified the distress related to the considerable burden and perceived responsibility to manage this all well, with little time and attention for self-care. Psychosocial interventions to assist family-based caregivers to develop adaptive skills to feel better prepared to actively engage in these healthcare partnerships and manage their unique challenges may address these challenges. No such interventions appear to be available currently for family-based carers. Yet evidence-based, cognitive-behavioural interventions that target resilience skills have shown promise in assisting health professionals to address the psychological distress associated with caregiving that may bear relevance to family-based caregivers [37]. It is likely that different sub-groups of family-based caregivers will require interventions that equip them with slightly different skills. Future work tailoring such interventions both in content and delivery mechanisms to better address family-based caregivers’ needs will be critical.

Our data also pointed to several considerations for the implementation of future interventions to ensure that these are accessible to caregivers. Of note, the same aspects of caregiving that contribute to distress and support needs (such as perceived responsibility and lack of time), may also form barriers to help-seeking and engaging in interventions [5]. As such, successful caregiver interventions are likely to be those which allow for flexible delivery, consistency and continuity in accessing the same healthcare professionals over time, and are designed with an openness to caregivers who may wish to engage and re-engage with supports at different points along the caregiving trajectory, and who need to ‘dip in and out’ of accessing support over time as needed. Future work developing and evaluating caregiver interventions also needs to characterise barriers and enablers relevant to the implementation of such programs in practice, for example, to determine when caregivers may be most likely to take up different types of support. Recent psycho-oncology intervention studies have highlighted that individuals’ time of greatest need is not necessarily the time when they will actively engage with supports, even when they express a desire and need for it [38, 39]. Developing such interventions with diverse caregiver populations through approaches such as co-design provides an avenue to ensuring suitable content and implementation approaches [40].

Alongside caregiver-focused interventions, oncology healthcare professionals may also benefit from the development of specific communicative skills to acknowledge and support the more clinical, healthcare-related aspects of caregivers’ roles. Comprehensive clinical guidelines now exist that can provide a useful scaffold to guide communication between triads of patients, caregivers and the healthcare-professional team, particularly in the context of psychosocial vulnerability, family-based complexity/conflict, and challenges in the cancer treatment trajectory [41, 42]. Such guidance is less well-developed in relation to communication with people from ethnic minority backgrounds and this is an area for development. Given the volume of care occurring out of hospital, upskilling not only oncology professionals but community-based broad healthcare teams may be valuable; general practitioners and community-based nurses and allied health professionals may be well-placed to provide an additional layer of support, separate to the person with cancer’s needs beyond the hospital environment [43].

### Limitations

Our data address an important gap in the literature around family-based caregivers, yet our findings should be considered in the context of several limitations. Our sample was relatively small, and predominantly female, and while we did purposely sample a subset of ethnic minority caregivers, it is likely that the experiences we have captured here do not reflect the full diversity of challenges experienced by family-based caregivers in the general population. There were no apparent points of difference between the data emerging from each data collection method in relation to the research questions, but it is possible that the use of two different methods shaped the resulting data. Our qualitative methodology richly captured the lived experiences and perceptions of our caregiver sample yet cannot address other perspectives including those of the health-professionals and systems with which these caregivers interacted. Future work is needed to examine how caregiver and health-professional perceptions may align or diverge, and to assess what clinical interventions may be appropriate and taken-up at various points of the caregiving trajectory.

## Conclusions

Family-based caregivers carry a great burden in terms of informal and supportive care for cancer patients across the cancer care trajectory. Unique challenges and sources of distress exist particular to the clinical- and healthcare-related roles caregivers undertake, and for caregivers from priority populations. Developing and delivering strategies to assist family-based caregivers in navigating these roles is a critical next step to enhance outcomes for patients, families, and the success of the healthcare partnership in cancer care. Such strategies must be developed with and for caregiver populations they seek to support, including priority groups.

## Data Availability

The datasets generated and analysed during the current study are not publicly available due to ethical approval restrictions that require consent from the participants to make their data available to other parties but are available from the corresponding author on reasonable request.
